# The Role of Hypoxia-Induced miR-210 in Cancer Progression

**DOI:** 10.3390/ijms16036353

**Published:** 2015-03-19

**Authors:** Kyvan Dang, Kenneth A. Myers

**Affiliations:** Department of Biological Sciences, University of the Sciences, 600 S. 43rd Str., Philadelphia, PA 19104, USA; E-Mail: k.dang@usciences.edu

**Keywords:** miR-210, hypoxia, microRNA, apoptosis, angiogenesis, cancer

## Abstract

Prolonged hypoxia, the event of insufficient oxygen, is known to upregulate tumor development and growth by promoting the formation of a neoplastic environment. The recent discovery that a subset of cellular microRNAs (miRs) are upregulated during hypoxia, where they function to promote tumor development, highlights the importance of hypoxia-induced miRs as targets for continued investigation. miRs are short, non-coding transcripts involved in gene expression and regulation. Under hypoxic conditions, miR-210 becomes highly upregulated in response to hypoxia inducing factors (HIFs). HIF-1α drives miR-210’s overexpression and the resultant alteration of cellular processes, including cell cycle regulation, mitochondria function, apoptosis, angiogenesis and metastasis. Here we discuss hypoxia-induced dysregulation of miR-210 and the resultant changes in miR-210 protein targets that regulate cancer progression. Potential methods of targeting miR-210 as a therapeutic tool are also explored.

## 1. HIF-1 Promotes Hypoxia-Induced Upregulation of microRNAs

Hypoxia is the condition of inadequate oxygen in the tissues. In response to hypoxic stress, cells alter DNA transcription in conjunction with oxygen-monitoring machinery, including Hypoxia-Inducible Factors (HIFs) that comprise the major components of hypoxia signaling pathways. HIF-1 is a heterodimer, composed of one oxygen-sensitive subunit, HIF-1α, and one oxygen-insensitive subunit, HIF-1β. Under normoxia, or normal oxygen levels, HIF-1β is active and HIF-1α is degraded by proteasomes with the help of prolyl hydroxylases, which function by hydroxylating specific proline residues (Pro402 and Pro564 in HIF-1α) that recruit proteasomes to carry out the oxygen-dependent degradation of the HIF-1α subunit [1,2]. In tumor microenvironments, the HIF-1α oxygen-dependent degradation pathway is halted, resulting in elevated levels of HIF-1α. Recent experimental evidence suggests that HIF-1α functions to specifically regulate cellular adaptation to the hypoxic condition through the recruitment of microRNAs (miRs) [3,4].

miRs are small, single-stranded noncoding RNAs that regulate the production of cellular proteins by either inhibiting messenger RNA (mRNA) translation, or by promoting the degradation of mRNA. This is achieved through a mechanism by which miRs utilize base pairing of their “seed” regions—nucleotides 2–8—with their target mRNA’s 3' untranslated region (UTR). Once base paired, the miR is targeted by the RNA-induced silencing complex (RISC), a complex composed of mature miRs and Argonaute proteins [5,6,7,8]. miRs can also bind sites other than the 3' UTR, such as those in mRNA coding regions downstream of the 3' UTR [9]. However, seed region binding is the most prominent mechanism because miRs’ affinity to mRNA is greater at the seed region than any other [10]. A single miR can affect the translation and activity of over 100 mRNAs, illustrating their wide influence over cellular processes [11].

Certain miRs are involved in regulating the HIF-1 pathway by targeting both upstream and downstream signaling molecules that function as oncogenes and/or tumor suppressors [7]. For example, downregulation of tumor suppressor miRs, such as miR-15 and miR-16, promotes carcinogenesis by upregulating anti-apoptotic proteins and insufficiently downregulating endogenous oncogenes. Similarly, overexpression of certain miRs can encourage tumor growth and cancer by downregulating endogenous tumor suppressors such as miR-199a and miR-20b, the latter of which downregulates HIF-1α during hypoxia by targeting its 3' UTR [6,12,13]. As a whole, miRs control at least 60% of protein-coding genes, and due to their roles in gene expression and their wide involvement in numerous cell processes, any malfunction in their own expression or regulation may pose significant problems—the most notable being the onset and progression of cancer [5].

**Figure 1:**
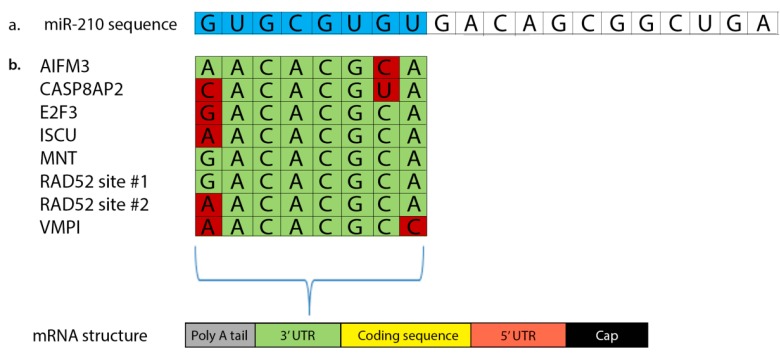
miR-210 binds to the 3' UTR of target mRNAs in order to modulate their transcription. (**a**) Entire mRNA sequence with seed region highlighted in blue; (**b**) 3' UTR of some of miR-210’s target mRNAs. Bases that successfully pair to miR-210’s seed region are highlighted in green. Bases that do not successfully pair are highlighted in red.

Of the miRs that are regulated by hypoxia through the HIF-1 pathway, the most responsive and influential miR is miR-210 [13,14]. miR-210’s seed sequence (6–8 nucleotides long) recognizes and binds complementary pairing sites on its targets’ 3' UTR (Figure 1). A limited amount of genes have been confirmed as bona fide targets, though the possibility of more targets exists [15]. Toward this end, *in silico* searches with prediction software, such as TargetScan and PicTar, have been performed to find potential miR-210 target mRNAs by matching 3' UTR to miR-210’s seed region. In addition to the 3' UTR, 5' UTR and coding regions of mRNA can also be used for matching [15], and these studies have revealed target pairing sites complementary to the seed region of miR-210 that include E2F3, RAD52, and MNT (Figure 1). Thus, a growing body of experimental evidence suggests that HIF-1α-mediated dysregulation of miR-210 directly modulates changes in mRNA transcription associated with altered control of the cell cycle, inadequate production of energy, cell death, and aberrant regulation of cell morphology, polarization and directed migration that characterize cancer pathology and metastasis.

## 2. miR-210 and HIF-1α Are Coordinately Regulated

Under normoxia, endogenous levels of miR-210 are maintained at very low levels [3]. Isoform specific stabilization of miR-210 is achieved by the binding of HIF-1α to the Hypoxia Responsive Element (HRE) present on its proximal promoter [13], in addition to an increase in miR-210 nascent primary transcript (pri-miR-210) [16]. HIF-1α promotes increased expression of miR-210, and miR-210 promotes the stabilization of HIF-1α, suggesting that a positive feedback loop is at work [17,18]. Moreover, because the level of miR-210 is dependent on the level of HIF-1α, the presence of elevated miR-210 in tissues has become a predictive marker for tumor hypoxia [6,19,20]. In this oxygen-dependent regulatory system, prolyl hydroxylases that normally function to induce the degradation of HIF-1α, are instead inhibited by miR-210, resulting in enhanced levels of HIF-1α [3]. In addition to prolyl hydroxylases, experimental evidence suggests that the miR-210 feedback loop functions by targeting and downregulating the Succinate Dehydrogenase Complex Subunit D (SDHD), another inhibitor of HIF-1α. In this way, miR-210-mediated repression of SDHD functions to promote HIF-1α stabilization and thereby further promotes miR-210 production to drive the positive feedback loop [3,21].

Additionally, HIF-1α-mediated regulation of miR-210 can occur through at least one oxygen-independent mechanism. An example of this effect is highlighted when a mutation in the von-Hippel Lindau (VHL) tumor suppressor leads to elevated levels of HIF-1α. VHL acts as an E3 ubiquitin ligase, selectively binding to hydroxyprolyl residues in HIF-1α and labeling affected proteins for proteasomal degradation. Thus, a mutation in VHL results in insufficient HIF-1α degradation, elevated HIF-1α levels, and a hypoxic response during normoxic conditions [13,22].

## 3. miR-210 Targets MNT and E2F3 to Promote Cell Cycle Progression

The MYC/Max/Mad network is a signaling pathway critical in the regulation of cell cycle and cell proliferation [18,23,24,25]. Under hypoxic conditions, stabilization of HIF-1α results in elevated cellular levels of miR-210 and activation of the c-MYC antagonist, MXI1, which inhibits the c-MYC oncoprotein and thereby halts the cell cycle at G_1_/S. Additionally, HIF-1α encourages the proteasomal degradation of c-MYC by disrupting its binding to target gene promoters [16].

Increased levels of miR-210 also promote the downregulation of Max’s Next Tango (MNT), a transcription factor and a member of MYC/Max/Mad network. Under normoxic conditions, c-MYC binds to Max, resulting in enhanced DNA binding and cell cycle progression, and MNT competes with c-MYC for Max binding. Maintaining an appropriate level of MNT-Max dimers is critical for the regulation of cell cycle entry and progression [16,23,26]. Under hypoxic conditions, miR-210 binds sequences in MNT’s 3' UTR (5' GCACAG 3') complementary to miR-210’s seed sequence (3' CGUGUC 5') and downregulates MNT transcription (Figure 1). MNT downregulation allows c-MYC to push cells through the cell cycle, suggesting that miR-210 indirectly activates c-MYC. In cases where these binding sites were disrupted, binding was unable to occur and MNT downregulation was halted. Additionally, knockdown of miR-210 with siRNA promoted increased MNT levels and resulted in arresting both the cell cycle and cell proliferation [16,20]. For example, in glioma stem cells, knockdown of miR-210 rescued G_0_/G_1_ cell cycle arrest via MNT-Max complex-dependent transcription repression [27].

Another miR-210 cell cycle regulatory target is the DNA transcription factor E2F transcription factor 3 (E2F3). The E2F protein family, which consists of both “repressive” and “activating” E2Fs, indirectly regulates DNA synthesis during G_1_/S phase of the cell cycle. E2F3 has a tumor suppressor association domain, which allows it to bind to tumor suppressor proteins and halt cellular growth [18,28]. An increase in miR-210 resulted in a decrease in E2F3 expression and promoted cell proliferation [29,30]. For example, an upregulation of miR-210 in ER-positive and ER-negative breast cancer cell lines resulted in increased proliferation, even in cells treated with the chemotherapeutic agent Tamoxifen [31]. In addition to proliferation, miR-210 dysregulation can result in abnormal control of the cell cycle that includes the formation of multipolar spindles and excessive amplification of centrosomes. Indeed, when cells overexpress miR-210, γ-tubulin, a microtubule nucleating factor and component of the centrosome, is also upregulated [32]. As a result, mitotic spindle assembly goes awry in miR-210 overexpressing cells, resulting in multipolar spindles, accumulation of cells at G_2_/M phase, and multi-nucleation.

## 4. Elevated miR-210 Disrupts Normal DNA Repair and Increases Genetic Instability

There is a greater instance of aneuploidy in miR-210-overexpressing cells than in normal cells [32]. Cells exposed to hypoxic environments are prone to genetic instability, aneuploidy, increased rates of DNA mutation, and they are compromised in their ability to repair aberrant DNA [33,34]. Current evidence suggests that inadequate DNA repair is primarily due to DNA repair genes that are downregulated in hypoxia and are commonly implicated in tumorigenesis, including *BRCA1*, *BRCA2*, *RAD51* and *RAD52*, members of the Homology-Dependent Repair (HDR) DNA repair pathway [33,34,35,36].

The HDR pathway repairs complex double-strand lesions through the recruitment of BRCA2 and RAD51 proteins to the site of the break, following recruitment of Replication Protein A (RPA) [36]. BRCA2 mediates the recruitment of RAD51 to RPA, and RAD51 subsequently repairs the double-strand break through a process involving the formation of nucleoprotein filaments [18,36,37,38]. In BRCA2-deficient cells, including certain types of breast cancer, RAD52 substitutes for the missing BRCA2 by recruiting RAD51 to the site of DNA breaks, suggesting that BRCA2 and RAD52 play very similar roles in the HDR pathway [36]. In hypoxic cells, miR-210 binds to two sites on the 3' UTR of the RAD52 mRNA (Figure 1) resulting in RAD52 mRNA degradation and rendering it unable to participate in DNA repair via the HDR pathway. Experimental evidence in support of this miR-210-induced effect showed that cultured cells overexpressing miR-210 exhibited higher levels of double-strand DNA breaks than did cells with normal levels of miR-210 [4], and that RAD52 levels were significantly lower in hypoxic cells [39]. Importantly, RAD52 was identified as the only DNA repair enzyme that is targeted for downregulation by miR-210 [33].

## 5. miR-210 Alters Normal Mitochondrial Function and Metabolism

Under hypoxia, cells can no longer acquire energy via the tricarboxylic acid (TCA) cycle, and must instead utilize the glycolytic, oxygen-independent pathway for energy production. In the case of metabolism under hypoxia, cells will stimulate glycolytic proteins including pyruvate dehydrogenase kinase (PDK1), lactate dehydrogenase A (LDHA), cytochrome *c* oxidase subunit 4-2 (COX 4-2), and the mitochondrial Lon protease. Additionally, hypoxia will induce cells to suppress proteins involved in mitochondrial respiration, including the iron-sulfur (Fe-S) cluster scaffold proteins, ISCU1 and ISCU2 [18,39,40,41]. ISCU1/2 play key roles in the assembly of Fe-S clusters that contribute to enzymatic energy production, and are also components of mitochondrial electron transport chain (ETC) complexes I, II, and III [4,39,40,41].

miR-210 targets and downregulates ISCU1/2 via their 3' UTRs, resulting in disruption of TCA metabolism and compromised electron transport and energy production [40]. This outcome is realized because miR-210-mediated disruption of ISCU1/2 promotes a shift from mitochondrial oxidative phosphorylation to increased rates of glycolysis coupled with lactic acid fermentation in the presence of oxygen, a phenomenon termed the Warburg effect [6,42]. Although lactic acid fermentation yields significantly less ATP than oxidative phosphorylation, the amount of ATP produced (2 ATP per molecule of glucose) is sufficient for cancer cell proliferation. Cancer cells may also prefer fermentation-associated glycolysis as a source of energy production because the production of lactate yields NAD^+^, another molecule that contributes to cancer cell proliferation [42]. Separate from miR-210’s effects on ISCU1/2, hypoxia-induced regulation of gene and/or protein targets of cellular metabolism and glucose transport may further contribute to this effect and tumorigenesis [4,6,39].

In addition to the disturbance of normal mitochondrial metabolism in cancer cells, increased levels of miR-210 have also been shown to contribute to aberrant mitochondrial membrane potential. This is thought to occur via miR-210-mediated downregulation of Succinate Dehydrogenase Complex subunit D (SDHD) complex II, which is a key component of ETC complex I and II. This effect has been experimentally visualized as structural irregularities including improper formation of mitochondrial cristae and a resultant reduction in mitochondrial membrane potential [40,43]. In addition to this effect, SDHD targeting also seems to stabilize HIF-1α, creating a positive feedback loop through the inhibition of prolyl-hydroxylases, responsible for HIF-1α degradation (discussed above). The delay in HIF-1α degradation leads to its stabilization and its subsequent up-regulatory effect on miR-210 [6,39,43,44].

## 6. Hypoxic Cells Evade Apoptosis through miR-210-Mediated Downregulation of AIFM3, CASP8AP2, and SIN3A

Hypoxic cells with elevated levels of miR-210 exhibit high viability due to their ability to evade apoptosis, suggesting a link between miR-210 and apoptosis during hypoxia. Comparison of apoptosis in cells depleted of miR-210 by siRNA revealed that apoptosis increased in hypoxic cells but not in normoxic cells, providing strong support for miR-210’s role in the regulation of hypoxia-induced apoptosis [45]. In addition, predicted miR-210 seed sites in apoptosis-related mRNA transcripts were investigated including the target protein Apoptosis-Inducing Factor Mitochondrion-associated 3 (AIFM3), a major player in caspase-dependent apoptosis [45]. AIFM3 localizes to the mitochondria and mediates the release of cytochrome *c* from the mitochondria to the cytosol [45,46,47]. miR-210-mediated downregulation of AIFM3 resulted in the inhibition of caspase-dependent apoptosis, while miR-210 knockdown resulted in the restoration of both cellular levels of AIFM3 protein and apoptosis [39,45].

Along with AIFM3, miR-210 has also been shown to target the 3' UTR of Caspase-8-Associated Protein-2, CASP8AP2, a component of a death-signaling complex implicated in activating Fas-mediated apoptosis. CASP8AP2 plays an integral role in the cleavage and activation of caspase-8, an initiator caspase that will activate apoptosis [45]. In cells where CASP8AP2 activity was downregulated by miR-210, apoptosis was non-existent in conditions that would normally trigger apoptosis, such as lethal anoxia and irradiation [48].

In hypoxic non-small cell lung carcinoma-derived cells (A549) overexpressing stable miR-210, caspase-3 activity was dramatically reduced. This resulted in decreased apoptosis in miR-210 upregulated cells compared to cells expressing endogenous levels of miR-210. Additionally, miR-210-overexpressing cells were able to proliferate in a gamma-irradiated environment, presumably by utilizing the miR-210-mediated inhibition of the caspase-dependent apoptotic pathway. Insensitivity to gamma-irradiation was found to be HIF-1-dependent, but independent of both p53 and position in the cell cycle [49]. Importantly, hypoxic HIF-negative cells were found to be less resistant to apoptosis than were HIF-positive cells, suggesting that the presence of HIF-1 plays a significant role in apoptosis evasion [46,49].

Similarly to both AIFM3 and CASP8AP2, miR-210 downregulation of SIN3A, a transcription repressor that forms a complex with histone deacetylase 1, results in decreased apoptosis and increased cell proliferation that has been associated with glioma formation and development [27,50]. A recent investigation found that therapeutic inhibition of miR-210 stimulated apoptosis and inhibited proliferation of a glioblastoma cell line. siRNA-mediated knockdown of SIN3A was sufficient to re-establish apoptosis evasion and unrestricted proliferation, highlighting SIN3A as another important miR-210 target that regulates cellular decisions to proliferate or to undergo programmed death [50]. Taken together, these studies support a model in which hypoxia-induced upregulation of miR-210 results in the targeting and aberrant regulation of key members of different apoptosis-related cell signaling pathways in order to drive uncontrolled cell proliferation and carcinogenesis.

## 7. miR-210 Promotes Angiogenesis and Metastasis

Angiogenesis, the process through which migratory endothelial cells form new blood vessels from pre-existing blood vessels, is a process responsible for providing tumor sustenance and for promoting cancer metastasis [51]. It has been shown that hypoxia can induce angiogenesis, suggesting the potential for miR-210-mediated regulation of endothelial cell angiogenesis [39]. Interestingly, miR-210-mediated effects on endothelial cell angiogenesis are related to miR210-induced effects on mitochondrial metabolism, where miR-210 overexpression promotes the switch from oxidative phosphorylation to lactic acid fermentation (described above). The miR-210-induced switch causes an increase in glucose transporters (such as GLUT-1), whose presence compensates for the reduction of glucose during glycolysis. GLUT-1 upregulation is followed by upregulation of Vascular Endothelial Growth Factor (VEGF) and Platelet-Derived Growth Factor (PDGF), generating an extracellular environment primed for angiogenesis [44]. Alternatively, miR-210 can control cellular levels of VEGF through the targeted regulation of receptor tyrosine kinase ligand Ephrin-A3 (EFNA3) and phospho-tyrosine phosphatase-1B (PTP1B). Both EFNA3 and PTP1B are negative regulators of VEGF [4]. miR-210-mediated down-regulation of EFNA3 or of PTP1B allows for increased expression of VEGF receptor 2 which promotes increased VEGF production and leads to angiogenic sprouting of capillary and tubular structures [4,6,7,18,39].

Recent investigations have shown that cancer cells grown in hypoxic conditions can communicate with target cells via exosomes, vesicles that contain proteins, RNA, miRs, and lipids [52,53]. The overwhelming presence of HIF-1α during hypoxia induces the production of exosomes and release of soluble factors, such as Tissue Inhibitor of Metalloproteinases-1 (TIMP-1). This increase in exosomes and TIMP-1 leads to an increase in miR-210 [53]. Exosomes may participate in intercellular communication by excreting miRs, such as miR-210. This communication is believed to occur between tumor and endothelial cells, as was shown when human leukemic cells (K562) were exposed to hypoxia [52,53]. This resulted in exosomes expressing elevated levels of miR-210 that were subsequently trafficked and released into the cytoplasm, resulting in the downregulation of EFNA3, subsequent upregulation of VEGF, and increased tubulogenesis within the target cells [52]. Similarly, an excess of TIMP-1 was found to trigger miR-210 upregulation via the PI3K/AKT pathway, resulting in high exosomal miR-210 and increased angiogenic tubulogensis [53].

In addition to increased angiogenic potential, cells overexpressing hypoxia-induced miR-210 possess significantly higher invasion potential than normoxic cells [54]. This is highlighted by the fact that metastasis is seen more frequently in cancer cells overexpressing miR-210 [55]. miR-210 affects both angiogenesis and metastasis by targeting and negatively regulating vacuole membrane protein 1 (VMP1), which normally functions by inhibiting cell migration and invasion. An overexpression of miR-210 resulted in a decrease in VMP1 mRNA and protein levels in various colorectal cancer cell lines and in hepatocellular carcinoma cells, promoting enhanced cellular migration and invasion, a result that is supported by studies of miR-210 knockdown in which cellular migration and invasion was inhibited [19,38,54,56].

## 8. miR-210 Promotes Senescence-Driven Carcinogenesis

Cellular senescence is an aging process defined by a loss of proliferative ability, and has been shown to result from age-related cellular insults including telomere shortening, oxidative stress, oncogene expression, and epigenetic modifications. [57,58]. Despite this, senescent cells are still able to metabolize normally, maintain normal morphology, and secrete soluble and insoluble factors. Recent studies suggest that miR-210 upregulation in senescent cells activates the Senescence-Associate Secretory Phenotype (SASP), a secretory process that modifies the microenvironment to establish carcinogenesis-promoting ECMs [57].

Under hypoxic conditions, senescent fibroblasts that were co-cultured with prostate cancer cells increased the rate of proliferation and enhanced the invasive nature of the prostate cancer cells by promoting epithelial-mesenchymal transition. This outcome was mediated through miR-210-directed effects on HIF-1α and a resultant increase in GLUT-1 and MCT4, glucose and lactose membrane transporters (respectively) capable of secreting growth factors to promote tumor growth [57]. Senescent fibroblasts are also capable of promoting an immunosuppressive microenvironment by stimulating monocyte recruitment and differentiation into pro-tumorigenic M2 macrophages. Additionally, miR-210 induced SASP from senescent fibroblasts can stimulate tumor vascularization and angiogenesis, resulting in significantly enhanced endothelial cell invasion and proliferation [57].

Additional evidence suggests that cellular insults that encourage senescence, such as hypoxia or DNA damage, are capable of generating a positive feedback loop to further induce miR-210 upregulation. This feedback loop, which is thought to involve the mTOR pathway, works by promoting continued miR-210-mediated activation of the cell senescence machinery [58].

## 9. miR-210 as a Tumor Suppressor

Although miR-210 promotes cell proliferation in many types of cancers, it has also been shown to play tumor suppressive roles in a small number of cancer cells including ovarian cancer, Esophageal Squamous Cell Carcinoma (ESCC) and Laryngeal Squamous Cell Carcinoma (LSCC) [59,60]. In ovarian cancer, miR-210 levels are maintained at relatively low levels due to gene copy loss. While the exact effects of this are unknown, evidence from other miR-210 tumor suppressor investigations provide insights into how the mechanism of tumor suppression is carried out [30,38].

In ESCC cell lines, for example, miR-210 expression levels were significantly lower than those found in control cell lines [60,61], while in LSCC cells, overexpression of miR-210 resulted in a dramatic increase in cells in G_1_/G_0_ and G_2_/M phases, and a decrease in cells in S phase, indicative of cell cycle arrest. The increased arrest was found to result from miR-210 binding and downregulation of Fibroblast Growth Factor Receptor-Like 1 (FGFRL1), a protein involved in cellular proliferation [59,60]. FGFRL1 tumor suppression function was also observed *in vivo* through experiments in which cells stably expressing miR-210 were subcutaneously transplanted into nude mice, resulting in significantly reduced tumor growth. This study identified an inverse correlation between tumor size and miR-210 expression level, and found that rescue with exogenous FGFRL1 was sufficient to promote tumor growth in the miR-210 expressing cells [59,60]. Thus, experimental evidence suggests that miR-210 can promote tumor progression or promote tumor suppression, depending on the cell type.

## 10. miR-210 as a Therapeutic Target

The miR-210 footprint is expansive, including targeted control of cell cycle progression, apoptosis, mitochondrial integrity, DNA repair, and endothelial cell angiogenesis, making this miR a high-interest target for prevention and treatment of tumor growth and metastasis. One current notion for implementing miR-210 in cancer prevention stems from recent experimental findings that, in cancer cells, levels of miR-210 are directly proportional to the hypoxia metagene (the “signature” gene expressed during hypoxia) and are directly proportional to HIF levels, supporting the potential utility of miR-210 as a biomarker that could be used to specifically measure hypoxia [4]. Besides specificity, perhaps the greatest advantage of this type of biomarker is non-invasiveness, because miR-210 levels can be easily detected in patient sera [7]. This technique has recently been experimentally explored in breast, pancreatic, and clear cell renal carcinoma cancers. In all three cases, increased levels of miR-210 were correlated with increased hypoxia and were also correlated with increased tumor tissue [14,62,63,64,65].

Increased levels of miR-210 expression are directly correlated with poor cancer patient outcomes. High miR-210 expression levels correlate with increased lymph node metastasis in pancreatic ductal adenocarcinoma patients [66]. Experimental studies of glioma revealed that high miR-210 levels were associated with increased tumor aggressiveness, and survivability [25,67]. Similarly, in adrenocortical carcinoma, elevated miR-210 expression was linked to increased tissue necrosis and proliferation [68]. This correlated relationship provides a potential opportunity to utilize miR-210 as an effective biomarker and prognostic tool. Biomarkers represent an exciting direction for therapeutic intervention with the potential for early and non-invasive cancer detection with the potential for early treatment and increased survivability [6,25,45,54,55,64].

**Figure 2:**
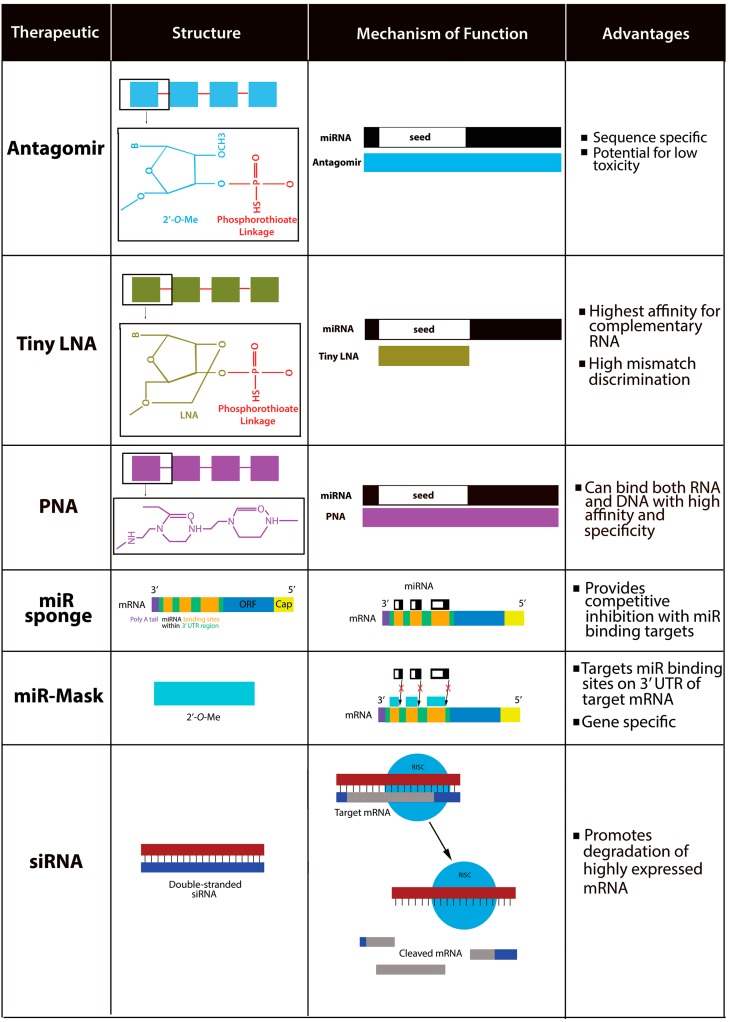
Structure, mechanism of function, and advantages of therapeutic strategies used to target miRNAs. For antagomirs, tiny LNAs, and PNAs, molecular structures of the first monomer and linkages are indicated within the black boxes.

While miR-210 has been the most extensively studied miR, there are a number of other miRs implicated in cancer, such as miR-21 and miR-122 [69], highlighting the importance of developing miR-targeting therapies for clinical treatments (Figure 2). Current clinical developments are focused on anti-miR oligonucleotides, or anti-miRs, including: antagomirs, miR-sponges, miR-masks, Locked Nucleic Acid (LNAs) probes, Peptide Nucleic Acid (PNA) probes, and siRNA, all of which could target oncogene-specific miRs [22,39,69]. In order for anti-miR therapeutic approaches to be beneficial and effective they must maintain efficient delivery, have optimized binding affinity and potency, and display limited toxicity and off-target effects, which usually occurs when specificity is compromised. Thus, specificity of treatment is paramount and must be achieved by modifying drug delivery and design strategies (see Section 11, below) [69,70].

Antisense miRs (anti-miRs) are competitive inhibitors of miRs, and are composed of oligonucleotides with chemically modified sugar, nucleotide base, inter-nucleotide linkages, or any combination of these modifications [69,71,72]. The appropriate combination of modifications is made to optimize binding affinity, nuclease resistance, and delivery of anti-miRs to its target miR [72,73]. For example, modifications made to the 2' position of the sugar moiety, such as 2'-*O*-methyl (2'-*O*-Me), 2'-*O*-methyoxyethyl (2'-MOE), and 2'-fluoro (2'-F), increase nuclease resistance and binding affinity of an anti-miR to its target miR [69,72,73]. Recently, a novel non-nucleotide modification was discovered and its use improves binding affinity and potency, and limits toxicity and both endonuclease and exonuclease-driven degradation [73]. The modification, *N*,*N*-diethyl-4-(4-nitronaphthalen-1-ylazo)-phenylamine (nicknamed “ZEN”), can be added at the 5' and 3' ends of oligonucleotides and requires no other modifications, such as LNA or PS linkages, to be effective. Several different drugs developed with ZEN and anti-miRs are being tested *in vivo*. Preliminary results show limited toxicity and heightened binding affinity [73]. Ultimately, effective anti-miRs will specifically recognize, bind, and prevent miR function in the cytoplasm while avoiding its own degradation [71,72].

Antagomirs, a specific type of anti-miR, are single-stranded RNA molecules conjugated with cholesterol. A single antagomir is 21–23 nucleotides in length and is complementary to a target miR [74]. An antagomir’s efficacy is determined by its structure. For example, successful antagomirs are at least 19 nucleotides in length and have phosphorothioate modifications, which offer protection against RNase activity [75]. Antagomirs function by binding to a mature miR to prevent miR-target mRNA binding, thereby inhibiting miR-mediated repression of the target mRNA [72]. Once the miR is intercepted by the antagomir, it is either formed into a duplex with the antagomir or degraded via a pathway independent of exonuclease activity and the RNAi pathway, though the exact details of this process in unknown [69,75]. For example, antagomir-122, an antagomir directed against miR-122, became the first established miR drug when it was shown to silence miR-122 in a sequence-specific fashion with low toxicity in mouse hepatocytes [76,77].

LNAs are anti-miRs that are water-soluble bicyclic nucleic acid analogues in which a methylene bridge connecting the 2'-O-atom and the 4'-C atom “locks” the ribose ring in place [69,70], resulting in increased potency, safety, and binding affinity [72,77]. LNAs have shown great promise as therapeutics because they display the highest affinity for complementary RNA [72], in addition to their high mismatch discrimination and the resultant increase in LNA specificity [69]. LNA oligonucleotides have been developed into their own anti-miR drug, termed tiny LNAs [78]. Tiny LNAs are composed of LNA-modified 8 monomers (8-mer) and a phosphorothioate backbone that are complementary to seed regions of miRs [72,78]. This design enables for miR seed family targeting with potent inhibition while at the same time providing for high specificity and low toxicity, such that if the tiny LNA binds sequences other than the seed, inhibition is absent [72,78]. For example, a LNA drug has been clinically test against hepatitis virus C with no evidence of viral resistance or toxic side effects [79].

PNAs are DNA analogs whose sugar-phosphate backbone is replaced by *N*-(2-aminoethyl) glycine subunits connected by peptide bonds [70,80], making them capable of binding both single-stranded RNA and DNA with high affinity and specificity without producing toxicity [70]. PNAs are also resistant to nucleases and proteases, increasing their appeal for use *in vivo* [81], and can be introduced to cells without the use of transfection reagents [70,80]. One such PNA conjugate, termed R8-PNA-a210, has been proven effective in targeting and inhibiting miR-210 in MDA-MB-231 breast cancer cells [80], supporting the utility of PNAs as therapeutic anti-miRs.

Additional types of anti-miRs include miR sponges, miR-masks, and siRNAs [7,69]. miR sponges are RNA transcripts that possess multiple tandem-binding sites in its 3' UTR to a target miR [69,70,82] and work by increasing the number of available complementary mRNA sequences to attract and “soak up” endogenous miRs by promoting competitive inhibition with *bona fide* miR binding targets. The effectiveness of miR sponges is comparable to that of LNA oligonucleotides [69]. Notably, a miR sponge targeting miR-9 has been shown to effectively inhibit metastasis in breast cancer. Despite its effectiveness, miR sponges pose potentially harmful side effects because their miR targets may share homogeneity with unintended targets, suggesting that modifications to this technology will likely be required in order to increase specificity and reduce off-target effects [82].

miR-masks are single stranded 2'-*O*-methyl-modified antisense oligonucleotides that are complementary to miR binding sites in the 3' UTR sequences of target mRNA [69]. miR-masks recognize and bind to miR binding sites on the 3' UTR of the target mRNA, thus preventing miR from binding at the same sites. This approach is gene specific and has been proven successful in inhibiting miR-430 repression of growth factor-β signaling pathways in zebrafish [69,70]. Finally, there are continuing efforts to generate siRNA that is specific to miR-210’s target genes. Like other anti-miRs, siRNA must be optimized in order to improve molecule biostability, specificity, and delivery. Modifications, such as LNA and 2'-*O*-methyl nucleotides, can be made in order to achieve sufficient efficacy [77,83] and have been successfully used to target the VEGF receptor 1 [7].

## 11. Strategies for Therapeutic Delivery

Methodology of therapeutic delivery is an essential topic when discussing the effectiveness and potential off-target effects of treatment. Considerations for therapeutic delivery include toxicity, harmful side effects, and potential routes to their amelioration. miRs and other nucleic acid-based cargo have been delivered using viral-based systems such as retrovirus, lentivirus, adenovirus, baculovirus, and Adeno-Associated Virus (AAV) [84,85]. Both retroviruses and lentiviruses have the ability to integrate transgenes into host genomes of dividing and non-dividing cells, though the risk of insertional mutagenesis is always present. To combat this, lentiviruses equipped with defective integrase, the enzyme required for proper integration into the host genome, have been used to maintain the transgene in its episomal form, without compromising its expression [84,85]. Lentiviruses have successfully delivered miR-15a and miR-16 into prostate cancer cells and have successfully induced tumor growth arrest, apoptosis, and regression [85].

Early attempts to utilize adenoviruses for transgene integration into dividing and non-dividing cell types have triggered fatal humoral and cellular immune responses. To counteract this effect, adenoviral vectors that contain only viral terminal repeats and no other viral components (*a.k.a.* “gutless” vectors) have been developed to evade immune detection, though this modification has been seen to reduce transgene expression [84]. Alternatively, baculovirus has been shown capable of effectively transducing mammalian chondrocytes and several types of stem cells (*i.e.*, neural, embryonic, induced pluripotent, and bone marrow) with limited pathogenicity, as long as the native insect vector is driven by a mammalian promoter [84,86]. Baculovirus-mediated miR delivery offers unique advantages because baculovirus cannot proliferate in mammalian hosts and because baculovirus degrades in the host over time, making this methodology a promising therapeutic approach [84]. Presently, AAV is the most encouraging viral system for gene therapy, as it can prolong transgene expression in its host and can transduce dividing and non-dividing cells [84,87]. AAV is non-pathogenic and thus poses a minimal threat to immune systems in humans. However, because a majority of the human population produces neutralizing antibodies against AAV, recombinant AAV (rAAV) approaches have been designed to deliver episomal miR-coding DNA into human cells. This technique is undergoing clinical evaluation for gene therapy treatments of cystic fibrosis and muscular dystrophy [87].

Synthetic tools provide a viable alternative to viral-based delivery systems and provide the advantage of user-controlled molecular composition, cargo load, manufacturing, and analysis, resulting in lower immunogenicity [70,88,89]. Several lipid-based drugs, such as lipoplexes (complexes in which miR is conjugated with empty liposomes) and polycationic liposome-hyaluronic acid nanoparticles have been tested *in vitro*, but their usage *in vivo* is limited due to high toxicity. Neutral lipid emulsion has been effective in delivering tumor suppressor miRs (miR-34a and let-7) to non-small lung cancer cells, resulting in a 60% reduction in tumor burden in mice [90]. However, because this approach preferentially targets to the lungs, its broader utility for miR delivery is likely limited [70].

Another promising synthetic miR delivery technique takes advantage of polyethylenimine (PEI)-based systems, which have an overall positive charge due to protonated amine groups, making PEI interactions with negatively charged polysaccharides on the cell surface and PEI endocytosis highly favorable [89,91]. Once inside the cell, PEIs promote the release of therapeutic miRs by soaking up protons that contribute to endosomal acidification [89]. PEI-based systems have successfully delivered DNA, ribozymes and siRNA into cells, and have been coupled to Rabies Virus Glycoprotein (RVG) to promote bypass of the blood brain barrier with reduced toxicity and increased targeted delivery. Therapeutic limitations of the PEI technology include maintenance of complex stability, *in vivo* distribution, cationic cytotoxicity, cell surface aggregation, and pharmacokinetics of miR delivery [89,92,93].

Poly(lactide-*co*-glycolide) (PLGA) derived nanoparticles overcome some of the limitations of the PEI approach and display characteristics of increased monodispersion and optimal charge ratio. Additionally, PLGA particles provide increased protection against miR degradation, high cargo limits and greater potential for modifications intended to improve pharmacodynamics. For example, PLGA particles have been coupled to miR-155 PNAs that were then effectively delivered to B-cell tumors with enhanced therapeutic efficacy [94,95]. Nanoparticle therapy for miR delivery has also been coupled to lipid modifications in order to achieve targeted therapeutic delivery. For example, interfering nanoparticles (iNOP-7), which use lysine dendrimers coupled to specific lipid chains, have been successfully used to downregulate apoB protein levels through the delivery of apoB siRNA into liver cells [96,97]. Additional adaptations of nanoparticle technology have focused on fine-tuning the specificity of miR delivery by using nanoparticle-aptamer modifications [98], as well as on/off approaches including pH-dependent miR release [99] and nanoparticle photo-activation [100] in order to induce localized miR delivery and thereby increase tissue specificity, limit toxicity, and reduce off-target effects.

## 12. Concluding Remarks

**Figure 3:**
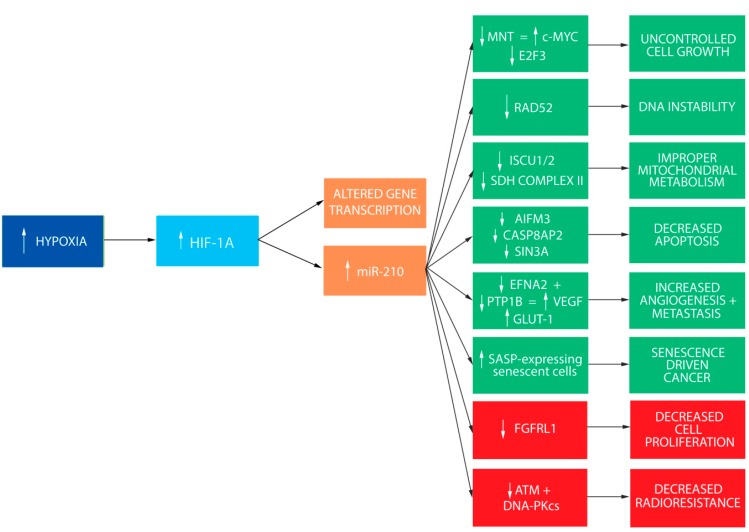
Effects of hypoxia-induced miR-210 on known mRNA targets. Green boxes indicate miR-210 targets that promote oncogenic activity. Red boxes indicate miR-210 targets that promote tumor suppression.

Hypoxia-induced miR-210 expression results in aberrant regulation of normal cell processes by altering the translation profile of a host of cellular proteins that drive cell proliferation, regulate DNA stability and mitochondrial metabolism, and trigger apoptosis and angiogenesis (Figure 3). To combat the effects of miR-210 and other miRs, several promising anti-miR-based therapeutic tools and methodologies for therapeutic delivery are being developed and tested to target and inhibit inappropriately upregulated miRs, though more studies are needed to prove their effectiveness *in vivo*. Increased investigations of the component miR family members and their targets, along with a clear understanding of how miR expression and function is regulated by the cell, may serve to promote new methodologies that can be used to therapeutically influence specific miR activities in order to curb the dysregulation of fundamental cellular processes and miR-driven disease.
